# Extrusion Process as an Alternative to Improve Pulses Products Consumption. A Review

**DOI:** 10.3390/foods10051096

**Published:** 2021-05-15

**Authors:** Mario Cotacallapa-Sucapuca, Erika N. Vega, Helayne A. Maieves, José De J. Berrios, Patricia Morales, Virginia Fernández-Ruiz, Montaña Cámara

**Affiliations:** 1 Nutrition and Food Science Department, Pharmacy Faculty, Complutense University of Madrid (UCM), Plaza Ramón y Cajal s/n, E-28040 Madrid, Spain; mariocot@ucm.es (M.C.-S.); erimavega@gmail.com (E.N.V.); helaynemaieves@gmail.com (H.A.M.); patricia.morales@farm.ucm.es (P.M.); vfernand@farm.ucm.es (V.F.-R.); 2 Escuela Profesional de Ingeniería Agroindustrial, Universidad Nacional de Moquegua, Prolongación Calle Ancash s/n, Moquegua 18001, Peru; 3 Faculdade de Nutrição, Universidade Federal de Pelotas, Rua Gomes Carneiro nº 01, Pelotas 96010-610, RS, Brazil; 4 E USDA-ARS-WRRC, 800 Buchanan Street, Albany, CA 94710-1105, USA; jose.berrios@usda.gov

**Keywords:** extrusion process, pulses, nutritional compounds, phytochemicals

## Abstract

The development of new food products obtained by extrusion processing has increased in recent years. Extrusion is used by the food industry to produce a wide variety of food products, such as ready-to-eat foods (e.g., snacks), among others. Pulses have also gained popularity as novel food ingredients in the formulation of a variety of food and food products, due to their high content of macro and micronutrients, and bioactive compounds that improve the nutritional and functional properties of the final food products. In this review, the impact of extrusion variables on proteins, carbohydrates, vitamins, phenolics and antinutritional compounds in pulses and pulse-based formulations are highlighted. Particularly, the impact of the specific mechanical energy. Also, the preservation, increase and/or reduction in those functional compounds, as a consequence of different extrusion processing conditions, are discussed.

## 1. Introduction

Extrusion (from the Latin word “extrudere”) means the action of pushing/forcing something out [1,2]. Currently, extrusion is regarded as a high-temperature short-time (HTST), versatile and modern food operation that converts agricultural commodities, usually in a granular or powdered form, into fully cooked food products [3]. It is a continuous processing technology that combines multiple unit operations such as transportation and compression, mixing, shearing, plasticizing, melting, cooking, denaturation, fragmentation, texturization, shaping, reactive extrusion, functionalization, separation, etc., in one single machine [4,5]. The unit operations carried out in the extruder are generated by one or two Archimedes screws rotating into the barrel, which induce thermal and mechanical stress to the feed material under processing, which promotes physical and chemical changes, and forces the product to flow through a small orifice, called “die” [4,6]. The promoted physical and chemical changes to the feed material involve complex changes in the food matrix, phytochemical composition and organoleptic properties, such as texture, colour and flavour [7,8]. 

One big advantage of the extrusion process is the capability to produce a wide range of finished food products with minimum processing time, using inexpensive raw material [3,9]. For this reason, extrusion is used by the food industry to produce a wide variety of highly consumed food products, such as some ready-to-eat food products or snacks [10,11,12,13]. These food products are traditionally based on starch raw materials, such as cereals and pseudocereals, since starch provides a cohesive mass that holds food components together [14].

Pulses are a type of leguminous crop. The Food and Agricultural Organization (FAO) defines “pulses” as those leguminous seeds harvested as dry grains; therefore, those leguminous seeds consumed as fresh vegetables (green peas, green beans, etc.), those mainly used for oil extraction (soybean and peanuts), and those crops used solely for sowing purposes (seeds of clover and alfalfa) are not considered “pulses” under the FAO definition [15]. Pulses represent an important source of gluten-free food for a healthy diet since they are an excellent source of protein, dietary fibre, minerals and vitamins. The world’s food production is estimated to increase by 70% due to an expected large increase in the world’s population. This food production increase would represent an increase of over 200 million tons of meat [16]. The increased consumption of meat has been recognized in several studies as a risk factor of common diseases such as type II diabetes, hypertension, dyslipidaemia, obesity and other cardiovascular diseases [17,18,19,20]. Pulses are recognized as a food with nutritional benefits and environmentally friendly crops, so their consumption should be encouraged in a daily diet. Based on the important contributions of pulses to the diet and the environment worldwide, the United Nations General Assembly (UNGA) declared 2016 as the International Year of Pulses [21].

The use of pulses as a healthy food source has been also recognized by the European Food Safety Authority [22], Agencia Española de Seguridad Alimentaria y Nutrición [23], U.S. Dietary Guidelines, National Heart [24], National Heart, Lung and Blood Institute [25], among other well-known institutions. They have recommended including pulses at least once a week in our diets, especially in substitution of animal protein, since the consumption of pulses four or more times a week were related with a 22% lower risk of coronary heart diseases and 11% of cardiovascular diseases [26,27].

Beans are the most consumed pulses around the world, followed by chickpeas and lentils [28,29]. Consequently, beans are also the highest produced pulses in the world, with 28,902,672 tonnes in 2019, followed by chickpeas with 14,246,295 tonnes, dry cowpea with 8,903,329 and lentil with 5,734,201 tonnes [30]. 

Review articles, such as Pasqualone et al. [31], have been published on the use of legumes in extruded products and the benefit of some extrusion parameters in the final products. The present review is aimed to relate the findings on the processing of pulses through extrusion technology as an interesting culinary process that improves the nutritional, functional and acceptability of pulses’ products. It also aims to describe the importance of extruded parameters, such as the specific mechanical energy and its impact on the final food products quality. Additionally, it gives a comprehensive and detailed compilation of the effects of extrusion on pulses’ macro and micronutrients, as water-soluble vitamins, and bioactive compounds. 

## 2. Food Extrusion Process and Variables 

The extrusion process involves many operational variables, such as barrel temperature, moisture content, screw speed and feed rate, etc. [32]. Combinations of different temperatures, pressures, and moisture may be used to create an unlimited range of product characteristics. For example, the density of individual pieces of an extrudate depends on the different pressure applied across the die [33]. The effects of the extrusion process parameters (temperature, screw speed, feed rate, screw configuration, die pressure and torque) and the raw material properties (e.g., proportion of ingredients, particle size, moisture content) on the quality of the extruded products have been studied by several researchers [34]. 

The structure of the extruded foods is a challenging scientific matter that has important implications for the food industry. Despite the significant progress in the modelling of the material flow in extruders and knowledge about the mechanical properties of cellular solid foods, the production of extruded foods with a targeted structure still relies on a trial-and-error approach. There is still no mechanical model capable of fully accounting for the influence of extrusion variables and material properties on expansion and final cellular structure [35].

Some important extruded parameters will be discussed afterwards to elucidate their impact on the final food products. 

### 2.1. Specific Mechanical Energy (SME)

SME is a scale-independent measure of the mechanical energy dissipated as heat inside the material under extrusion. SME has been defined by some authors as the amount of mechanical energy or work input from the driver motor into the raw material being extruded, which is produced due to the friction generated between the screw and the product [11,36,37]. SME is a system parameter of the extrusion process used to try to establish a relationship between the processing variables and the properties of expanded extrudates, such as density, expansion ratio, solubility and degree of gelatinization.

The process parameters of the feed moisture, screw speed, barrel temperature and raw material properties have a great effect on the SME. Therefore, SME provides a good characterization of the extrusion process [11].

Once the raw material is introduced into the barrel, it is subjected to the rotational motion of the screw(s) and compacted, and submitted to more or less intense shear according to the screw geometry and rotation speed. SME has been used to evaluate the work exerted by the screw on the food matrix.

### 2.2. Mechanism of Specific Mechanical Energy

In a typical extrusion cooking process, the main processing variables considered are the ingredient feed rate (FR), water addition (WA), screw speed (SS) and barrel temperature (BT). While the variables generated by the process are barrel melt moisture content (MC) calculated from the WA and feed moisture (FM), percentage motor power consumed as indicated by the motor load (ML), pressure at the die (DP), and the product melt temperature (TM). Therefore, the extrusion cooking process can be viewed as a 4-input x 4-output process, from a systems perspective [38]. 

Other authors concurred that the food extrusion process involves multiple input and output systems resulting in complex interactions [39]. To facilitate system identification for the subsequent control design, it is desirable to reduce the dimensionality of the system to be controlled. Since the variables desirable to be controlled in a product (chemical) are strongly related to the process (physical) variables that can be controlled, it is necessary to find a correlation matrix between the product variables and the process variables. To simplify the multiplicity of the variables, the use of SME as the main process variable has been proposed [40].

A systematic analysis of the extrusion process of puffed corn snack products revealed that the SME and screw speed (SS) were a desirable pairing of variables to measure and manipulate, respectively, for regulating the extrude density [38]. Other authors have indicated that an increase in the screw speed causes greater friction and shearing between the materials under extrusion, leading to an increase in SME [11]. Moreover, the SME dissipated during shearing is transformed into thermal energy, increasing the temperature of the material, and modifying the physical and chemical properties of the resulted extruded product [32]. This information indicates that SME is a good indicator of the degree of cooking in materials subjected to the extrusion process. 

## 3. Changes on Food Composition Due to Extrusion Process 

Extrusion is a thermo-mechanical process that induces many chemical and structural transformations in the phytochemical composition of the product, as complex formation between amylose and lipids, degradation reactions of vitamins and pigments, among others [41]. Extrusion has also the advantage of increasing protein and starch digestibility, solubilizing dietary fibre, inactivating thermolabile toxins and some antinutritional factors, as well as undesirable enzymes such as lipoxygenases and peroxidases [13,42]. Moreover, it was reported that some bioactive compounds, such as the total phenolic compounds and α-galactosides were not greatly affected [42]. However, these results were highly conditioned by the extrusion conditions and percentage of the selected raw material in the mix, used as the feed material for extrusion [43,44]. 

### 3.1. The Influence of Extrusion Process on Pulse Proteins

Pulses are a good source of functional proteins. The most representative protein class is the globulins, which comprise around 70% of the total pulse’s proteins, and according to their sedimentation coefficient can be categorized as 7S and 11S, or vicilin and legumin, respectively. 

The protein content in pulses varies from 17 to 30%, which represents twice the value of the protein amount present in cereals. Moreover, pulse and cereal proteins are considered complementary, since pulses are rich in the amino acid lysine but relatively low in sulphur amino acids (methionine and cysteine), which are higher in cereals [44,45,46].

From the total pulse protein content, 4 to 20% are albumins that contain enzymatic proteins, such as protease inhibitors, amylase inhibitors, and lectins; while, prolamins and glutelins represent 1–3 and 10–20% of the total protein, respectively [47]. Prolamins are characterized as having a high amount of proline and glutamine, while the glutelins are characterized for the presence of methionine and cysteine [45,46,47,48,49].

The high-temperature, shear, and stress conditions that the extrusion process imparts on the treated material promotes denaturation of the proteins, leading to the loss of their ternary and quaternary structure, a decrease in disulphide bonds and an increase in protein solubility [31,50,51,52]. 

Also, due to the high processing temperature and the natural presence of certain amino acids and sugars in the material, the Maillard reaction takes place, which significantly affects the characteristics of the products, particularly their colour, aroma, and nutritional value [53,54,55]. Additionally, another detrimental product of the Maillard reaction is acrylamide, which is classified as a group 2a carcinogen. More specifically, acrylamide is produced mainly under conditions of high temperature and low moisture due to the reactions between the reducing sugars and asparagine. This reaction can also occur with other free amino acids, such as glutamine, arginine, cysteine, and aspartic acid, but in those cases only traces of acrylamide were determined [56,57]. To minimize the acrylamide formation in the material under extrusion, an increase in feed moisture or the injection of CO_2_ during processing can promote a gradual decrease in the acrylamide content in the extrudate. Other reported potential strategies to reduce the acrylamide content in extruded products are the addition of amino acids, such as cysteine or lysine, asparaginase enzyme, the control of processing temperatures, or the addition of pulses [56,58]. Galani et al. [59] observed that the inclusion of chickpea in cereal-base formulations, heated at 160 °C for 20 min, reduced acrylamide formation while it improved the nutritional value of the formulated flours. Moreover, Tuncel et al. [60] reported that the addition of 5% of pea flour to a bread formulation containing wheat bran and whole bran, resulted in a decrease of 57% and 68% in the acrylamide levels.

Various authors highlight that extrusion technology is an effective processes to improve the protein quality and digestibility of pulses and pulse-based extrudates. Table 1 summarises the most relevant studies of the effect of extrusion conditions on the protein content of pulse extrudates. 

High extrusion temperature conditions were reported to have the effect of decreasing the amount of protein in different extruded pulses (peas, chickpeas, faba beans and kidney beans), at barrel temperatures over 180 °C; while no significant changes were observed at barrel temperatures close to 140 °C [61,62]. Most importantly, extrusion cooking had a positive effect on pulse extrudates by improving their water solubility and water absorption indexes, inactivating antinutritional components, and increasing their protein digestibility. Therefore, the resulted improved pulse extrudates were considered adequate to be added as a functional ingredient (usually in powder form) in the preparation of bread, soups, and creams [61,62,63].

When comparing extrusion, traditional cooking, and baking technologies in the processing of different types of beans and lentils, extrusion technology was most effective to preserve the protein content in the final product. Extrusion also improved their protein solubility and in vitro digestibility. Additionally, extrusion promoted an increase in amino acid content, particularly those sulphur-containing amino acids. The reported findings allowed to conclude that the extrusion process is a suitable process to improve amino acid availability and protein digestibility [64,65]. It has been demonstrated that protein denaturation and a reduction in enzyme inhibitors, due to extrusion processing, improved the digestibility of proteins. However, the extent of the improvement depended on the food matrix and the type of pulses under consideration, among other parameters [63,66]

The use of different food ingredient sources to develop acceptable and quality extruded products has been the focus of the most recent investigations. Arribas, et al. [43] reported the improvement of the nutritional quality of rice-based ready-to-eat food with bean and whole carob fruit (*Ceratonia siliqua* L.). They concluded that the developed extruded product had a balanced protein profile and improved protein digestibility, compared to the extruded product without bean addition.

The response surface methodology (RSM) has been used by some researchers to investigate the best conditions for product optimization of the extrusion process. Guldiken et al. [67], using RSM to optimize the extrusion process of a mixture of chickpea and barley flours (60:40), were able to obtain a product with great protein quality under the extrusion conditions of 160 °C barrel temperature, 18% feed moisture and 320 rpm screw speed. 

### 3.2. Carbohydrates Content in Extruded Pulse Product

Pulses contain between 60–65% carbohydrates, a bit lower than in cereals, which contain between 70 and 80% [44]. The main storage carbohydrate in pulses is starch, and other carbohydrates found in pulses are monosaccharides (ribose, glucose, galactose, and fructose), disaccharides (sucrose and maltose), oligosaccharides (mostly α-galactosides) and other polysaccharides [3,12,27,68].

Starch is the main source of carbohydrate in the human diet. Starch represents 30–40% of the daily energy intake [69], and is one of the most available and economical organic food materials on Earth [70]. Starch granules are composed primarily of amylose and amylopectin, of which proportions vary depending on the cultivar botanical origin. The amylose/amylopectin ratio has been found to be a determinant of the functional properties of starch-based materials, such as mechanical properties, oxygen permeability, water binding capacity, gel property, viscosity etc., and gives starch its typical properties (solubility, cooking time, gelatinization, etc.) that determine the suitability of a type of starch for a particular final use [10,71]. Starch with an amylose/amylopectin ratio of approximately 1:3 or 1:4 provides a suitable ingredient to obtain expanded and crispy products [72].

A prerequisite for obtaining a cohesive dough is the presence of a gel former food ingredient. Therefore, in this regard, free amylose, pre-gelatinized starch, or pre-gelatinized waxy starch can be used as a gel former and promote a cohesive mass to improve the functional properties of the extruded products [14]. Additionally, some other authors have reported that modified starch or hydroxypropylated starch with a high amylose content produce films with a high mechanical strength, flexibility and transparency. They also indicated that the stability of hydroxypropylated starch was highly dependent on the amylose content [73].

Starch is an essential food ingredient widely used in food extrusion. During extrusion, the starch–water mixture becomes melted by solid friction, viscous dissipation and heat conduction, causing a dense material consisting of starch and protein. The dense material is then forced through a matrix where the water vapour trapped in the melt expands the material giving a porous structure, called solid foam [74]. The pore shape and volume in the foam is determined by the natural ability of amylose and amylopectin polymer chains to assemble into an organized supramolecular nanoscale lamellar structure, within the starch granule consisting of crystalline and amorphous regions [70]. The extrusion conditions of the temperature profile, shear rate, and residence time during treatment, influence simultaneously the starch behaviour and its properties. Moreover, several authors have reported that the effects of the starch structural properties and starch modification, during the extrusion treatment on the product properties, were monitored via die pressure, motor torque, mean residence time and specific mechanical energy (SME) [75]. This finding goes along with the report of Pardhi et al. [41] who indicated that during extrusion cooking, the starch undergoes many physical, chemical, and structural transformations. 

Regarding the effect of the extrusion process on the carbohydrate fraction in pulses, variability in various results is reported. Several authors determined a significant increase in the total available content of carbohydrates after extrusion cooking, Cardoso-Santiago and Arêas [76] reported a significant increase of 5.4% in chickpeas (200 rpm of single-screw extruder at 130 °C and 13% feed moisture). Alonso et al. [77] observed an increase of 1% and 3.2% in extruded samples of pea and kidney bean, respectively, after extrusion-processing conditions of 100 rpm screw speed, 25% moisture barrel and a temperature of 150 °C. Morales et al. [78] reported a significant increase, around 5–17% in the extruded lentil flours processed on a twin-screw extruder at 160 °C die temperature, 500 rpm speed twin screw and 17% feed moisture. Arribas et al. [10] reported a 1 to 9% increase in the extrudates formulated with 20% beans and 5% whole carob, as well as with 40% beans with 10% whole carob, respectively, extruded at 125 °C die temperature, 900–950 rpm screw and 10.0–12.8% feed moisture. The determined increase may be due to an increase in the carbohydrate availability present inside the cell generated by the diffusion of the solvent inside the matrix, leading to a subsequent increase in the specific surface area trough cell rupture and higher cell wall porosity [78,79,80].

On the other hand, some authors reported a decrease in the amount of total carbohydrates. Berrios et al. [3] determined that the total available carbohydrates in the extruded lentil, chickpea and dry pea flours decreased in all the extruded flours compared to their corresponding raw counterpart. However, they also indicated that only the decrease in the total carbohydrates determined in the chickpea samples were considered significative. The temperature, moisture and screw speed conditions applied during extrusion are known to have major physicochemical modifications. Therefore, the reported decrease in the total carbohydrates may be explained by a macromolecular degradation of polysaccharides during processing, promoting the release of monosaccharides. Also, the extrusion process could have hydrolysed oligosaccharides, possibly through the breaking of the 2→1-furanosidic bonds in sucrose and raffinose, promoting the formation of lower molecular weight sugars. Likewise, the monosaccharides behaviour can also vary during processing, as is summarized in Table 2 [3,81].

#### 3.2.1. Dietary Fibre and Their Modification under Extrusion Process

Currently, dietary fibre is defined as “food material, particularly plant material, that is not hydrolysed by enzymes secreted by the human digestive tract but that may be digested by microflora in the gut” [83]. Dietary fibre can be classified according to different criteria, as chemical, analytical, or physiological. However, the most generally accepted classification is based on their solubility in water systems under physiological conditions. Dietary fibre represents two groups of fibres, insoluble and soluble dietary fibre. Soluble dietary fibre (SDF) is readily fermented in the large intestine by the microflora, and it consists mostly of natural gel-forming fibres like pectins, gums, mucilages, inulin, fructans, some hemicelluloses and non-digestible oligosaccharides. Due to the ability of the forming gels, the addition of SDF can maximize the viscous characteristic of foods and delay the digestion time of nutrients. Insoluble dietary fibre (IDF) contributes to the promotion of the laxation effect, through the increase in faecal bulk. The main components of IDF are cellulose, some hemicellulose, lignin, arabinoxylan and resistant starch [27,84,85,86,87]. 

The amount of TDF in pulses varies between 3–30% depending on different factors such as agronomic, genetic, seed maturation, and processing conditions. The amount of IDF represents the major fraction, which varies between 4 and 28% of the TDF [85,88]. In 2011, Oomah et al. [44] published a recompilation of six studies carried out between 1997 and 2003 where they reported DF contents of 23–32, 18–22, 18–20, 14–26 (%) for bean, chickpea, lentil, and dry pea, respectively. Later, Cámara et al. [84,85] compiled and reported data of the DF content in pulses from different countries. They reported values of DF content ranging between 16.12 to 26.20, 6.5 to 29.80, 20.35 to 27.60, and 3.31 to 29.70 (g/100 g), for chickpea, lentils, common beans, and dry green peas, respectively.

Resistant starches (RS) are starch molecules that resist digestion and in this sense, they function similarly to DF. Even though RS are not always included as part of the dietary fibre fractions in foods, their physical property behaviour is very similar to that exhibited by IDF. Studies indicated that RS is fermented in the gut, producing short-chain fatty acids (SCFAs) such as acetate, propionate, butyrate, and lactate, in addition to methane and carbon dioxide. SCFAs are a good source of energy preferred for the proliferation of colonocytes, and they help in the prevention of growing abnormal cells (by lowering intracellular pH), increasing blood flow, improving the availability of some minerals such as calcium and magnesium, and the absorptions of iron [27,44,84,89,90].

Many studies have reported an increase in the soluble dietary fibre (SDF) content, as a consequence of extrusion processing. The increase in SDF determined in the final extrudates was related to the presence of oligosaccharides, which resulted from the breakdown of the polysaccharides glycosidic bonds [91], the presence of new glucans branches resistant to amylase hydrolysis generated by the transglycosidation reaction between starch or fragments of starch and anhydro-compounds [92,93], the release of gums and mucilage’s by the breakdown of interactions (hydrogen bonds, hydrophobic interactions or covalent bonds) between these polysaccharides and phenolic compounds [94,95], and the breaking of covalent and non-covalent linkages from the carbohydrate–protein complex [94,96]. The reported increase in SDF was followed by a decrease in IDF, since most of the previously mentioned reactions involve the conversion of IDF to SDF. Conversely, an increase in IDF had also been reported in other studies, as a consequence of extrusion processing. The authors justified those results by an increase in the resistant starch content, due to starch gelatinization during processing [91,97], and to polysaccharide–protein complex formations (through the Maillard reaction), since these complexes present similar physico-chemical behaviour to IDF and are generally measured as lignin [91,92,94,98].

Additionally, several studies have reported that the extrusion processing conditions of moisture and temperature had a direct effect on the dietary fibre content of various food materials, as detailed in Table 3. Stojceska et al. [99] observed an increase in DF in the gluten-free products made with a variety of fruits and vegetables, Zhang et al. [100] reported that SDF in extrude oat bran had higher yields, improved rheological behaviour and functionality compared with those of SDF in unprocessed oat bran. Jing and Chi [101] made use of RSM to determine the optimum extrusion conditions for the processing of soy waste. They determined that the screw extrusion conditions of 180 rpm increased the amount of SDF in the soy waste extrudates. Similarly, Chen et al. [102] reported an increase in SDF in soybean waste using the blast extrusion process.

Some authors have stated that the effect of the extrusion processing conditions on the content of DF depends, to a large extent, on the type of food matrix analysed [42,104]. Also, the literature indicates that there are limited studies regarding the effect of the extrusion processing conditions on the DF of pulses, compared to those reports in cereal products [99,105,106] as well as in other food products [107,108]. Some specific studies on extruded lentil, dry pea, chickpea, and common bean, reported a partial or complete redistribution of IDF to SDF with only small changes in TDF [3,31,42,78,81]. Additionally, studies on the effect of extrusion processing on the DF content of some ready-to-eat products enriched with pulses, demonstrated that the improvement in the DF content in those products was dependent on the amount of pulses contained in the mixes. That is, the higher the inclusion of pulses in the mixes, the higher the content of DF, independent of the processing conditions [43,103].

#### 3.2.2. α-Galactosides and the Impact of Extrusion Process on Oligosaccharides

The α-galactosidic bonds in the oligosaccharides (raffinose, stachyose, verbascose and ciceritol) present in pulses cannot be metabolized by the human intestinal tract due to the lack of an α-galactosidase enzyme. Therefore, these carbohydrates remain undigested in the human intestine, where they are fermented by intestinal bacteria generating flatulence [109,110]. Despite the indicated undesirable effect caused by the indigestible oligosaccharides, it has been reported that the oligosaccharides can increase the bifidobacteria population in the colon, and in turn they act on stimulating the immune system and reducing digestive disorders [28,111]. Extrusion was reported to decrease the oligosaccharides content in the extrudates from different whole pulse flours, processed under high temperatures and frictional forces, which break down the oligosaccharides into more digestible carbohydrate components [12,77,112]. A similar effect to oligosaccharides has also been reported in the extruded samples of rice enriched with bean or pea flours, as detailed in Table 4 [43,82].

The observed decrease in the oligosaccharides content resulted in an increase in their carbohydrate components. Some authors have indicated that the increase in the carbohydrates components could be due to the modification of the food matrix structure, such as the release of oligosaccharides linked to other macromolecules, cell wall breakage, and the improvement in the porosity and specific surface area, which facilitates the extraction of the component soluble sugars. A particular study reported that the decrease in oligosaccharides in extruded black bean flour may be due to the breakdown of the (2→1) furanosidic bond, due to the high temperatures used in the extrusion process [81]. Verbascose is a penta-oligosaccharide conformed by the linkage of stachyose and raffinose, consequently the hydrolysis of verbascose generates the release (increase) of its component sugars [12,80].

### 3.3. Vitamins Content in Extruded Pulses Products

Vitamins are organic elements present in most natural foods that are essential for physiological functions, but when these are thermally/cook processed, particularly by the extrusion process, a relevant amount of the vitamins are lost [113]. 

As vitamins differ greatly in their structure and chemical composition, their stability during extrusion is also variable [7]. The extent of the vitamins degradation depends on their exposure to certain parameters encountered during food processing and storage, such as moisture, temperature, light, oxygen, time, and pH [57]. 

Water-soluble vitamins are more liable to be destroyed by their exposure to high temperatures encountered during the extrusion process, than fat-soluble vitamins. The fat-soluble vitamins most sensitive to extrusion cooking are vitamin A and vitamin E, while vitamins D and K are quite stable [7,113]. Provitamin A (carotenoids) and tocopherols (with vitamin E activity) are not stable in the presence of oxygen and heat. The main factor contributing to the reduction in β-carotene during extrusion is thermal degradation [57].

Table 5 shows some examples of the effect of extrusion processing conditions on the vitamin content of several pulses. Morales et al. [78] observed that extrusion caused a significant decrease (83–94%) in the tocopherols content in extrudates formulated with red chief lentils (*Lens culinaris* L.). Similarly, Ciudad-Mulero et al. [42] reported a reduction of 81.5–92% in the tocopherols content of extruded lentils. Zieliński et al., [114] determined a significant decrease (63–94%) in the tocopherols and tocotrienols content in extruded cereals. In addition, they observed that the proportion of tocotrienols to tocopherols increased after extrusion cooking, indicating that tocotrienols were the main residual isomers of vitamin E. 

As previously indicated that the water-soluble vitamins present at a lower heat stability than the fat-soluble vitamins. Riaz et al. [113] reported that the most sensitive water-soluble vitamins during heat processing were vitamin C, thiamine (B_1_) and folic acid (B_9_). Camire et al., [116] corroborated that thiamine and pyridoxine were the most thermolabile of the water-soluble vitamins and that their levels decreased linearly with increased processing temperatures. Brennan et al., [117] reported that high extrusion temperatures and low feed moisture promoted great degradation of ascorbic acid. Similar processing conditions were reported by Gulati and Rose [118] to also degrade folic acid. Conversely, water-soluble vitamins, such as B_2_, B_6_, B_12_, niacin, pantothenic acid, and biotin, were determined to be largely stable during the high-temperature conditions of the extrusion process [57]. 

Moreover, Athar et al., [115] concluded that some water-soluble vitamins were not highly affected by extrusion temperature and other extrusion conditions, when processed using a short-barrel extruder with a low retention time. Since, after processing a mixture of corn and pea, the extruded product presented a 61, 60, 70 and 18% retention of thiamine, niacin, riboflavin, and pyridoxine, respectively. Thus, an effective way to minimize the loss of vitamins in extruded food materials is to process them under mild extrusion temperatures and a short retention time.

Furthermore, Singh et al., [57] proposed nutritional enrichment options in order to maintain good levels of vitamins in the final processed and stored products. The proposed nutritional enrichment options included specific vitamin compounds and/or specific chemical forms with better stability, as well as enrichment with extra value amounts of nutrients. Also, the post-extrusion addition of vitamins.

### 3.4. Phenolic Compounds in Extruded Pulse Products

Phenolic compounds are bioactive secondary plant metabolites synthesized during the normal development of the plant, or generated as a response to stress conditions in order to protect the plant against predators, pathogens, parasites or UV radiation. Phenolic compounds have an antioxidant capacity that neutralizes free radicals to prevent damage due to oxidative stress. Therefore, phenolics are effective anti-inflammatory compounds and have antimicrobial properties, both of which are associated with health benefits. A large number of authors have agreed that phenolic compounds could help in the prevention of cardiovascular diseases, inflammation and diabetes [119,120,121,122,123].

The phenolic compounds present in major quantities in pulses are flavonoids, tannins, and phenolic acids. Among them, phenolic acids are the most representative of the phenolic compounds. There are the following two classes of phenolic acids: hydroxybenzoic acids and hydroxycinnamic acids (chiefly coumaric, caffeic and ferulic acids) [124]. In 2007, Xu and Chang [125] determined the presence of phenolic compounds in different pulses, and they reported that lentils presented good values of gallic acid (6.56 mg GAE g^−1^ for phenolic compounds) and catechin (1.30 mg CE g^−1^ for flavonoids and 5.97 mg CE g^−1^ for condensed tannin). They also indicated that lentil was the pulse with the highest amounts and better antioxidant activity, followed by red kidney and black beans. Additionally, they concluded that the content of phenolic compounds in pulses depended on genetic, environmental factors, and the type of pulse.

Even though phenolic compounds are excellent antioxidant agents, heat causes the degradation of phenolics, mainly by decarboxylation. Some authors have reported that the high-temperature conditions used in extrusion cooking promoted a decrease in the content and stability of the phenolic compounds [126]. In addition to temperature (the most influential parameter), some studies demonstrated that a low moisture content in the feed also has a detrimental effect on the phenolics content of the extruded products [127]. Furthermore, high-temperature extrusion conditions can lead to polymerization of some phenolic compounds [128,129].

However, despite the reported decrease in the phenolic compounds induced by extrusion cooking, the percentage decrease in antioxidant activity determined in the final extrudates was variable [128,130]. Extrusion may disrupt/break covalent bonds in a high molecular weight polyphenolic complex, generating other phenolic compounds, as well as releasing combined phenolics bound to dietary fibre and/or proteins [129,131].

Brennan et al. [117] and Nayak et al. [132] reported that the effect of extrusion in phenolic compounds was affected by many variables, such us the type of phenolic compound, the extrusion processing conditions, and/or the characteristics of the food matrix. Sensoy et al. [133] reported that the extrusion of buckwheat (*Fagopyrum esculantum* Moench) flour, processed at a temperature of 170 °C, did not generate any change in the antioxidant activity of phenolics, while roasting at 200 °C for ten minutes promoted a decrease in the antioxidant activity of the processed product. They concluded that the functionality of active compounds could be maintained by the optimization of time and processing temperature. Moreover, Korus et al. [134] demonstrated that the type of cultivar highly affected the phenolic content determined in the processed products. They reported that the total phenolic content in a common bean (*Phaseolus vulgaris* L.) variety, rawela (dark red) was 14% higher than in other two bean varieties, tip-top (black-brown) and toffi (Cream), after processing them under the same conditions. 

Ciudad-Mulero et al. [42] analysed the phenolic content of different extruded lentil flours formulated with nutritional yeast, processed at a die temperature of 140 °C and 160 °C, with a moisture feed of 17% and screw speed of 500 rpm. They reported a decrease in the total phenolic content in all samples, but especially in those samples extruded at the higher temperature of 160 °C and the higher inclusion of nutritional yeast (12 and 16%) in the formulations. Conversely, the antioxidant activity determined in most of the extruded sample flours increased significantly.

### 3.5. Antinutritional Compounds in Extruded Pulse Products

The nutritional value of pulses may be negatively affected by the presence of anti-physiological substances that can disrupt the process of assimilation in some nutrients, reduce the nutritive value of pulse-based foods, and can even be toxic. Those substances are generally called antinutritional compounds (ANCs) and have been categorized into two groups. The first group is represented by protein ANCs, which includes antimicrobial proteins such as lectins or agglutinins, and protease enzyme inhibitors like trypsin and chymotrypsin inhibitors. The second group is represented by non-protein ANCs, which includes saponins and other glycosides, alkaloids, phytic acid or phenolic compounds such as tannins [135,136,137,138,139,140]. Other studies, however, have demonstrated that the use of ANCs in specific amounts can help in the prevention of different diseases like cancer and coronary diseases [141,142,143].

Inositol hexaphosphate (IP6) or phytic acid present in pulses, are considered as antinutritional compounds for their capacity to effect complex minerals, such as zinc and calcium, which hinder their absorption. Nevertheless, less phosphorylated forms of inositol phosphate (IP5 and IP4) have demonstrated to have beneficial antioxidant activity [12,43]. Moreover, less phosphorylated forms of inositol phosphate have shown to have a negligible complex capacity for impairing mineral absorption [144]. The main extrusion processing conditions of temperature, moisture and screw speed have demonstrated to promote the partial hydrolysis of IP6 into less phosphorylated forms, rendering extruded products with the subsequent potential beneficial effect of the less phosphorylated forms IP4 and IP5. In this regard, Alonso et al. [77] reported the findings of a study using pea and kidney bean flours, processed in a twin-screw extruder at a screw speed of 100 rpm, 25% feed moisture, and die temperatures of 150 °C and 155 °C. The contents of inositol phosphate, in both of the extruded flours, were determined using high-performance liquid chromatographic (HPLC). The analytical results showed a decrease in the IP6 content of 27.67% and 26.81% for extruded pea and kidney bean flour, respectively, and a significant increase in the IP5 and IP4 content. 

El-Hady and Habiba [61] reported that other extrusion processing conditions can also influence the amounts of IP in the final extrudates. They extruded faba bean, pea, chickpea and kidney bean flours at two barrel temperatures (140 °C and 180 °C) and two feed moisture contents (18% and 22%). They determined, in all of the extruded flours, a reduction in phytic acid (IP6), especially in the samples processed at a temperature of 180 °C and 22% moisture content. A similar finding was reported by Rathod and Annapure [66], who processed a mixture of 40% lentil and 60% rice flour under the extrusion conditions of 70, 95 and 120 °C barrel temperature and 16, 20 and 24% feed moisture content. They evidenced the highest reduction in phytic acid (IP6) at the processing conditions of 120 °C and 24% moisture. Batista et al. [63] quantified the amount of phytic acid present in the raw and extruded flours of two hard-to-cook beans of different cultivars. The beans were extruded at a die temperature of 150 °C, screw speed of 150 rpm and feed moisture of 20%. The raw flours of the two bean cultivars (Pontal and Grafite) had phytic acid contents of 8.18 and 11.26 mg/g (dwb), respectively, which indicated the influence of cultivar in their composition. After the extrusion process, the phytic acid content in the flour of Pontal and Grafite showed a reduction of 17% and 26%, respectively. 

Also, the composition of the initial food mix influences the content of IP in the final extrudate. Morales et al. [12] reported that lentil flour enriched with wheat bran, NUTRIOSE^®^ or apple fibre presented a higher reduction in IP6 than lentil flour enriched with corn fibre, even though the determined decrease was not significant. Similarly, Arribas et al. [43] extruded a mix of bean flour (20–40%) with different percentages of rice (50–80%) and carob fruit (5–10%). The authors reported that the extrusion process significantly reduced the phytic acid content (10%) and significantly increased the content of less phosphorylated forms (16–70%). More specifically, there was a significant increase in the IP4 (16–52%) and IP5 (30–70%) forms of phytic acid. They also concluded that the reduction in phytic acid (IP6) content was higher in samples without carob fruit and less in the formulations containing a higher amount of pulses in the mixes.

Regarding tannins, these compounds have the tendency to form a complex with proteins and minerals, preventing the proper absorption of these nutrients. It has been demonstrated that the extrusion process considerably reduces the amounts of tannins in different pulses such as faba bean, pea, chickpea, and kidney bean, and that with higher processing temperatures and moistures, the reduction in tannins is greater [61,145]. The reduction in tannins may occur by alteration of their molecular structure or by changes in their chemical reactivity during extrusion processing. Also, it has been proposed that a reduction in tannins may occur during processing due to an increase in tannin polymerization, which may lead to a reduction in their extractability [62,77].

The effects of the extrusion process on the inactivation of the enzyme inhibitors trypsin, chymotrypsin, and α-amylase (Table 6), partially or totally, have been well established by Alonso et al. [77] in pea and bean flours extruded at a barrel temperature of 150 °C, speed screw of 100 rpm and feed moisture of 25%. Morales et al. [12] also reported similar findings in different lentil-based flour formulations extruded at a die temperature of 160 °C, speed screw of 500 rpm and feed moisture of 17%. Moreover, the results of a study carried out by Arribas et al. [82] also corroborate the previously reported findings. They extruded a mixture of rice flour enriched with bean and carob flours or pea and carob flours, processed under the following conditions: barrel temperature of 125 °C, screw speed of 900–950 rpm and feed moisture of 10%. Under these extrusion conditions, the protease inhibitors were fully inactivated. The decrease in enzyme/protease inhibitors may be due to their heat-sensitive nature, which in combination with low extrusion moisture promoted the total elimination of these compounds. Morales et al. [12] indicated that the limited elimination of trypsin inhibitors, observed in some cases, could be due to the presence of SH–SH bonds in these enzyme inhibitors and to the crystallized starches formed, acting as protectors.

All of the presented studies provide evidence that extrusion processing promoted the production of products without or with reduced amounts of inositol hexaphosphate (IP6), tannins and enzyme inhibitors, avoiding the undesirable effects of these compounds. While it also maintained or increased the amounts of the less phosphorylated forms of phytic acid, IP5 and IP4, improving the beneficial effects and the nutritional value of the developed products [66,146]. Further studies on the sensory properties (texture, colour, and flavour) of extruded pulse products should be considered, as the success of those products rely on consumer’s acceptance [43]

## 4. Conclusions

Extrusion is a continuous process that combines different unit operations, including mixing, baking, kneading, shear, moulding and forming. The heat transfer to the food material during processing has an impact on the extent of the physicochemical changes and the quality of the final extruded products. Therefore, the scale-independent measure of mechanical energy, known as specific mechanical energy (SME), is considered a relevant system parameter used to control the extrusion processing operations, which has a great impact in the preservation of valuable amounts of macro and micronutrients (water-soluble vitamins) in the extrudates. It also may impact the preservation of bioactive compounds such as polyphenols, dietary fibre and alpha-galactosides. Other benefits of extrusion processing include the increase in protein and starch digestibility, inactivation of undesirable enzyme inhibitors, as well as a reduction in the amount of inositol phosphates and tannins in pulses and other food materials.

This review showed that extrusion processing, under different processing conditions, induces chemical and physical changes that favour the bioavailability of important nutrients, and bioactive compounds in pulses and pulse-based extruded flours.

## Figures and Tables

**Table 1:** Effect of different extrusion processing conditions on the protein content of pulses and pulse-based extrudates.

Food Matrix	Extrusion Conditions	Effect: In the Amount	Effect: Protein In Vitro Digestibility	Reference
Faba bean (*Vicia faba* L.)	Single; ss: 250 rpm; bt: 140 °C; m: 18%; ft: 100 °C sc: 4:1	Insignificant decrease	Increase in vitro	[61]
	Single; ss: 250 rpm; bt: 180 °C; m: 18%; ft: 100 °C; sc: 4:1	Insignificant decrease	Increase in vitro	
	Single; ss: 250 rpm; bt: 140 °C; m: 22%; ft: 100 °C; sc: 4:1	Insignificant decrease	Increase in vitro	
	Single; ss: 250 rpm; bt: 180 °C; m: 22%; ft: 100 °C; sc: 4:1	Significative decrease	Increase in vitro	
	Twin; ss: 400–600 rpm; bt: 30–50 °C, 70–90 °C and 100–120 °C; m: 0.7–1.2 kg/h liquid feed rate	Small increase	High improvement	[64]
Pea seeds (*Pisum sativum* L.)	Single; ss: 250 rpm; bt: 140 °C; m: 18%; ft: 100 °C; sc: 4:1	Insignificant decrease	Increase in vitro	[61]
	Single; ss: 250 rpm; bt: 180 °C; m: 18%; ft: 100 °C; sc: 4:1	Significant decrease	Increase in vitro	
	Single; ss: 250 rpm; bt: 140 °C; m: 22%; ft: 100 °C; sc: 4:1	Insignificant decrease	Increase in vitro	
	Single; ss: 250 rpm; bt: 180 °C m: 22%; ft: 100 °C; sc: 4:1	Significant decrease	Increase in vitro	
Chickpeas (*Cicer arietinum* L.)	Single; ss: 250 rpm; bt: 140 °C; m: 18%; ft: 100 °C; sc: 4:1	Insignificant decrease	Increase in vitro	
	Single; ss: 250 rpm; bt: 180 °C; m: 18%; ft: 100 °C; sc: 4:1	Insignificant decrease	Increase in vitro	
	Single; ss: 250 rpm; bt: 140 °C; m: 22%; ft: 100 °C; sc: 4:1	Insignificant decrease	Increase in vitro	
	Single; ss: 250 rpm; bt: 180 °C; m: 22%; ft: 100 °C; sc: 4:1	Insignificant decrease	Increase in vitro	
Chickpea +40% barley bland	Twin; ss: 320 rpm; bt: 150, 160 °C; m: 18%	The higher the temperature, the higher was the increase in the amount of protein digestibility		[67]
Kidney beans (*Phaseolus vulgaris* L.)	Single; ss: 250 rpm; bt: 140 °C; m: 18%; ft: 100 °C; sc: 4:1	Insignificant decrease	Increase in vitro	[61]
	Single; ss: 250 rpm; bt: 180 °C; m: 18%; ft: 100 °C; sc: 4:1	Significant decrease	Increase in vitro	
	Single; ss: 250 rpm; bt: 140 °C; m: 22%; ft: 100 °C; sc: 4:1	Insignificant decrease	Increase in vitro	
	Single; ss: 250 rpm; bt: 180 °C; m: 22%; ft: 100 °C; sc: 4:1	Insignificant decrease	Increase in vitro	
Kidney bean carioca (pontal)	Single ss: 150 rpm; bt: 150 °C; m: 20%; cr: 3:1	Insignificant increase	Increase in vitro (72.3%)	[63]
Kidney bean black (grafite)	Single; ss: 150 rpm; bt: 150 °C; m: 20%; cr: 3:1	Insignificant increase	Increase in vitro (84.5%)	
Kidney bean black	Twin; ss: 400–600 rpm; bt: 30–50 °C, 70–90 °C and 100/120 °C; 0.7–1.2 kg/h liquid feed rate	Insignificant increase	Increase in the true and in vitro	[64]
Kidney bean navy	Twin; ss: 400–600 rpm; bt: 30–50 °C, 70–90 °C and 100/120 °C; 0.7–1.2 kg/h liquid feed rate	Insignificant increase	Increase in the true and in vitro	
Kidney bean pinto	Twin; ss: 400–600 rpm; bt: 30–50 °C, 70–90 °C and 100/120 °C; 0.7–1.2 kg/h liquid feed rate	Insignificant increase	Improvement in the true and in vitro	
Red kidney bean	Twin; ss: 400–600 rpm; bt: 30–50 °C, 70–90 °C and 100/120 °C; 0.7–1.2 kg/h liquid feed rate	Insignificant increase	Improvement in the true and in vitro	
Rice (80–60%) kidney bean (*Phaseolus vulgaris* L.) (20–40%)	Twin; ss: 900–950 rpm; bt: 125 °C; m: 2.5–3.2 kg/h	Small increase	Great improvement	[10]
Red lentil (*Lens culinaris* L.)	Twin; ss: 650 rpm; bt: 30–50 °C, 70–90 °C and 100–120 °C; m: 0.8 kg/h	Small increase	Improvement in the true and in vitro	[65]
Green lentil (*Lens culinaris* L.)	Twin; ss: 650 rpm; bt: 30–50 °C, 70–90 °C and 100–120 °C; m: 0.8 kg/h	Small increase	Improvement in the true and in vitro	

Twin or single: type of screw; ss: screw speed; bt: barrel temperature; ft: feed temperature; m: moisture; cr: compression ratio.

**Table 2:** Effect of extrusion processing conditions on sugars of different pulse extrudates.

Matrix	Extrusion Conditions	Sugars					Reference
		Sucrose	Maltose	Fructose	Galactose	Ribose	
Bean (*Phaseolus vulgaris* L.)	Non-Extruded	20.8 ± 0.05	3.5 ± 0.28 *	-	0.1 ± 0.01	-	[81]
	Twin, ss: 200 rpm; bt: 160 °C; m: 20%	20.1 ± 0.02	4.2 ± 0.24	-	0.5 ± 0.01	-	
Bean (*Phaseolus vulgaris* L.) + 0.4% NaHCO_3_	Non-Extruded	19.2 ± 0.10	4.0 ± 0.02	-	0.6 ± 0.01	-	
	Twin, ss: 200 rpm; bt: 160 °C; m: 20%	16.3 ± 0.10	3.5 ± 0.11	-	1.0 ± 0.01	-	
Dry pea (*Pisum sativum* L.)	Non-Extruded	6.47	1.91	1.24	7.22	5.21	[3]
	Twin; ss: 500 rpm; bt: 160 °C; m: 17%	12.99	n.d.	n.d.	n.d.	n.d.	
Chickpea (*Cicer arietinum* L.)	Non-Extruded	7.94	01.68	1.56	1.84	0.58	
	Twin; ss: 500 rpm; bt: 160 °C; m: 17%	10.03	01.77	1.45	0.66	0.62	
Lentil (*Lens culinaris* L.)	Non-Extruded	6.97	00.39	0.85	n.d.	3.05	
	Twin; ss: 500 rpm; bt: 160 °C; m: 17%	6.01	01.33	1.03	n.d.	1.36	
Lentil (*Lens culinaris* L. Medik)	Non-Extruded	10.22 ± 0.54	3.09 ± 0.18	-	-	-	[12]
	Twin; ss: 500 rpm; bt: 160 °C; m: 17%	12.82 ± 0.63	5.51 ± 0.46	-	-	-	
Rice (80%) + Pea (*Pisum sativum* L.) (20%)	Non-Extruded	4.76	-	-	-	-	[82]
	Twin; ss: 900 rpm; bt: 125 °C; m: 2.50 kg/h	9.44	-	-	-	-	
Rice (60%) + Pea (*Pisum sativum* L.) (40%)	Non-Extruded	7.97	-	-	-	-	
	Twin; ss: 950 rpm; bt: 125 °C; m: 2.50 kg/h	14.46	-	-	-	-	
Rice (80%) + Bean (*Phaseolus vulgaris* L.) (20%)	Non-Extruded	8.65 ± 0.15	-	-	-	-	[43]
	Twin; ss: 900 rpm; bt: 125 °C; m: 2.50 kg/h	10.27 ± 0.08	-	-	-	-	
Rice (60%) + Bean (*Phaseolus vulgaris* L.) (40%)	Non-Extruded	12.44 ± 0.15	-	-	-	-	
	Twin; ss: 950 rpm; bt: 125 °C; m: 2.50 kg/h	17.84 ± 0.16	-	-	-	-	

Values are expressed in mg/g; * maltose + inositol; twin or single: type of screw; ss: screw speed; bt: barrel temperature; m: moisture; n.d.: not detected.

**Table 3:** Extrusion processing conditions effect on the dietary fibre content (soluble, insoluble and total) content of extruded products.

Food Matrix	Extrusion Conditions	Extrusion Effect (%)			Reference
		SDF	IDF	TDF	
50% rice, 12.5% milk powered, 12.5% potato starch, 12.5% corn starch, 12.5 soya	Twin; ss: 275–350 rpm; bt: 80–150 °C; m: 12%; ft: 17–23 kg/h			↑16.76–112.6	[99]
20% rice, 12.5% milk powered, 12.5% potato starch, 12.5% corn starch, 12.5 soya, 30% apple	Twin; ss: 275–350 rpm; bt: 80–150 °C; m: 12%; ft: 17–23 kg/h			↑2.08–34.9	
20% rice, 12.5% milk powered, 12.5% potato starch, 12.5% corn starch, 12.5 soya, 30% cranberry	Twin; ss: 275–350 rpm; bt: 80–150 °C; m: 12%; ft: 17–23 kg/h			↑7.3–33.2	
20% rice, 12.5% milk powered, 12.5% potato starch, 12.5% corn starch, 12.5 soya, 30% carrot	Twin; ss: 275–350 rpm; bt: 80–150 °C; m: 12%; ft: 17–23 kg/h			↑10.02–28.32	
20% rice, 12.5% milk powered, 12.5% potato starch, 12.5% corn starch, 12.5 soya, 30% beetroot	Twin; ss: 275–350 rpm; bt: 80–150 °C; m: 12%; ft: 17–23 kg/h			↑2.93–23.27	
20% rice, 12.5% milk powered, 12.5% potato starch, 12.5% corn starch, 12.5 soya, 30% teff	Twin; ss: 275–350 rpm; bt: 80–150 °C; m: 12%; ft: 17–23 kg/h			↑139.2–190.8	
Oat bran	Twin; ss: 150 rpm; bt: 100–160 °C; m: 10–30%; ft: 18 kg/h	↑11.24–59.55			[100]
Soy waste	Twin; ss: 180 rpm; bt: 115 °C; m: 31%	↑10.45	↓10.43	↑0.03	[101]
Soy wasted	Twin; ss: 150 rpm; bt: 170 °C; ft: 30 kg/h	↑27.4		↓25.7	[102]
Black bean (*Phaseolus vulgaris* L.)3	Twin, ss: 200 rpm; bt: 160 °C; m: 20%	↑0.89	↓1.12		[81]
Black bean (*Phaseolus vulgaris* L.) + 0.2%NaHCO_3_	Twin, ss: 200 rpm; bt: 160 °C; m: 20%	↑2.91	↓3.54		
Lentil (*Lens culinaris* L.)	Twin; ss: 500 rpm; bt:160 °C; m: 17%	↑0.44	↓4.66	↓4.22	[78]
Lentil (*Lens culinaris* L.) + wheat bran + apple fibre	Twin; ss: 500 rpm; bt:160 °C; m: 17%	↑0.63	↓0.56	↑0.07	
Lentil (*Lens culinaris* L.) + wheat bran + NUTRIOSE^®^	Twin; ss: 500 rpm; bt:160 °C; m: 17%	↑0.04	↓4.03	↓3.9	
Lentil (*Lens culinaris* L.) + apple fibre + NUTRIOSE^®^	Twin; ss: 500 rpm; bt:160 °C; m: 17%	↑0.02	↓1.2	↓2.38	
Lentil (*Lens culinaris* L.) + apple fibre + corn fibre	Twin; ss: 500 rpm; bt:160 °C; m: 17%	↑0.11	↓0,84	↑0.08	
Lentil (*Lens culinaris* L.)	Twin; ss: 500 rpm; bt: 140 °C; m: 17%	↑1.59	↑4.92	↑6.5	[42]
Lentil (*Lens culinaris* L.)	Twin; ss: 500 rpm; bt: 160 °C; m: 17%	↑0.35	↑3.23	↑3.57	
Broken rice + bean grains (“Carioca” type)	Twin; ss: 277 rpm; bt: 80 °C; m: 14%			↓14.4	[103]
80% rice + 20% bean (*Phaseolus vulgaris* L.)	Twin; ss: 900–950 rpm; bt: 125 °C; m: 10–12%	↑2.39	↓5.53	↓3.16	[43]
60% rice + 40% bean (*Phaseolus vulgaris* L.)	Twin; ss: 900–950 rpm; bt: 125 °C; m: 10–12%	↓3.34	↓5.89	↑2.56	

SDF: soluble dietary fibre; IDF: insoluble dietary fibre; TDF: total dietary fibre; twin: type of screw; ss: screw speed; bt: barrel temperature; ft: feed temperature; m: moisture; ↑: increase; ↓: decrease.

**Table 4:** Effect of processing conditions on oligosaccharides content in pulse extrudates.

Food Matrix	Extrusion Conditions	Oligosaccharides (mg/g)						Reference
		Galactinol	Ciceritol	Galactopinitol	Verbascose	Raffinose	Stachyose	
Pea (*Pisum sativum* L.)	Non-Extruded				14.0	9.2	23.0	[77]
	Twin; ss: 100 rpm; bt: 150 °C; m: 25%				18.7	9.7	18.5	
Kidney bean (*Phaseolus vulgaris* L.)	Non-Extruded				2.4	6.4	41.4	
	Twin; ss: 100 rpm; bt: 150 °C; m: 25%				1.9	2.2	33.6	
Dry pea (*Pisum sativum* L.)	Non-Extruded	-	n.d.	-	-	15.64	20.19	[3]
	Twin; ss: 500 rpm; bt: 160 °C; m: 17%	-	n.d.	-	-	8.16	15.29	
Chickpea (*Cicer arietinum* L.)	Non-Extruded	-	2.686	-	-	6.05	14.14	
	Twin; ss: 500 rpm; bt: 160 °C; m: 17%	-	2.880	-	-	7.54	18.85	
Lentil (*Lens culinaris* L.)	Non-Extruded	-	2.249	-	-	12.08	n.e.	
	Twin; ss: 500 rpm; bt: 160 °C; m: 17%	-	2.372	-	-	2.22	11.80	
Pea (*Pisum sativum* var. *laguna* seeds)	Non-Extruded	-	-	-	6.0 ± 0.03	11.4 ± 0.04	23.6 ± 0.07	[112]
	Single; ss: 60 rpm; bt: 129 °C; m: 40 L/h	-	-	-	5.3 ± 0.02	10.2 ± 0.05	20.4 ± 0.05	
	Single; ss: 60 rpm; bt: 135 °C; m: 35 L/h	-	-	-	4.8 ± 0.03	7.4 ± 0.04	17.5 ± 0.06	
	Single; ss: 60 rpm; bt: 142 °C; m: 25 L/h	-	-	-	4.7 ± 0.02	7.6 ± 0.04	17.3 ± 0.03	
Lentil (*Lens culinaris* L. Medik)	Non-Extruded	0.32 ± 0.01	23.05 ± 0.59	1.82 ± 0.05	16.54 ± 0.41	2.72 ± 0.20	22.42 ± 1.74	[12]
	Twin; ss: 500 rpm; bt: 160 °C; m: 17%	1.36 ± 0.16	26.65 ± 0.77	2.84 ± 0.18	13.64 ± 0.46	4.22 ± 0.26	24.67 ± 0.76	
Rice (80%) + Pea (*Pisum sativum* L.) (20%)	Non-Extruded	0.27	n.d.	-	7.44	3.06	4.85	[82]
	Twin; ss: 900 rpm; bt: 125 °C; m: 2.50 kg/h	17.87	0.34	-	10.97	6.36	7.47	
Rice (60%) + Pea (*Pisum sativum* L.) (40%)	Non-Extruded	0.37	0.59	-	14.0	3.99	8.83	
	Twin; ss: 950 rpm; bt: 125 °C; m: 2.50 kg/h	8.84	1.05	-	22.01	8.51	11.89	
Rice (80%) + Bean (*Phaseolus vulgaris* L.) (20%)	Non-Extruded	2.53 ± 0.10	0.78 ± 0.04	-		2.84 ± 0.13	8.55 ± 0.40	[43]
	Twin; ss: 900 rpm; bt: 125 °C; m: 2.50 kg/h	2.84 ± 0.11	5.44 ± 0.07	-		9.46 ± 0.35	10.30 ± 0.43	
Rice (60%) + Bean (*Phaseolus vulgaris* L.) (40%)	Non-Extruded	3.58 ± 0.03	1.14 ± 0.05	-		3.87 ± 0.04	11.82 ± 0.23	
	Twin; ss: 950 rpm; bt: 125 °C; m: 2.50 kg/h	3.81 ± 0.10	8.92 ± 0.07	-		12.21 ± 0.24	20.91 ± 0.13	

Twin or single: type of screw; ss: screw speed; bt: barrel temperature; ft: feed temperature; m: moisture; n.e.: not evaluated.

**Table 5:** Effect of extrusion processing conditions on the vitamins content of pulse and cereal extrudates.

Vitamin	Food Matrix	Extrusion Conditions	Effect (mg/100 g)		Reference
			No-Extruded	Extruded	
Tocopherols	(Wheat cv. Almari)	Twin, ss: 500 rpm, bt: 120 °C, m: 20%	2.78	0.862	[114]
		Twin, ss: 500 rpm, bt: 160 °C, m: 20%		0.623	
		Twin, ss: 500 rpm, bt: 200 °C, m: 20%		0.933	
			
	(Barley cv. Gregor)	Twin, ss: 500 rpm, bt: 120 °C, m: 20%	1.873	0.354	
		Twin, ss: 500 rpm, bt: 160 °C, m: 20%		0.382	
		Twin, ss: 500 rpm, bt: 200 °C, m: 20%		0.31	
			
	Rye (cv. Dankowskie Złote)	Twin, ss: 500 rpm, bt: 120 °C, m: 20%	2.778	0.608	
		Twin, ss: 500 rpm, bt: 160 °C, m: 20%		0.885	
		Twin, ss: 500 rpm, bt: 200 °C, m: 20%		0.872	
			
	Oat (cv. Sławko)	Twin, ss: 500 rpm, bt: 120 °C, m: 20%	1.159	0.732	
		Twin, ss: 500 rpm, bt: 200 °C, m: 20%		0.099	
	Lentil (*Lens culinaris* L.)	Twin, ss: 500 rpm, bt: 160 °C, m: 17%	6.17 ± 0.25	1.02 ± 0.17	[78]
		Twin; ss: 500 rpm; bt: 140 °C; m: 17%	2.55 ± 0.01	0.30 ± 0.01	[42]
		Twin; ss: 500 rpm; bt: 160 °C; m: 17%		n.d.	
Thiamine	Oats	Single; ss: 0.012 kWhkg^−1^, bt: 152 °C, m: 7%	0.11	23 *	[115]
	Maize	Single; ss: 0.095 kWhkg^−1^, bt: 130 °C, m: 7%	0.09	62 *	
		Single; ss: 0.095 kWhkg^−1^, bt: 152 °C, m: 7%		44 *	
		Single; ss: 0.095 kWhkg^−1^, bt: 160 °C, m: 7%		62 *	
	Maize + dried green peas	Single; ss: 0.095 kWhkg^−1^, bt: 152 °C, m: 7%	0.22	61 *	
	Pea (*Pisum sativum* L. var. laguna)	Single; ss: 60 rpm, bt: 129 °C, m: 40L/h	0.196 ± 0.012	0.104 ± 0.009	[112]
		Single; ss: 60 rpm, bt: 135 °C, m: 35L/h		0.100 ± 0.008	
		Single; ss: 60 rpm, bt: 142 °C, m: 25L/h		0.089 ± 0.005	
Riboflavin	Oats	Single; ss: 0.012 kWhkg^−1^, bt: 152 °C, m: 7%	0.06	100 *	[115]
				
	Maize	Single; ss: 0.095 kWhkg^−1^, bt: 130 °C, m: 7%	0.04	100 *	
		Single; ss: 0.095 kWhkg^−1^, bt: 152 °C, m: 7%		86 *	
		Single; ss: 0.095 kWhkg^−1^, bt: 160 °C, m: 7%		100 *	
	Maize + dried green peas	Single; ss: 0.095kWhkg^−1^, bt: 152 °C, m: 7%	0.08	70 *	
	Pea (*Pisum sativum* L. var. laguna)	Single; ss: 60 rpm, bt: 129 °C, m: 40 L/h	0.102 ± 0.004	0.096 ± 0.006	[112]
		Single; ss: 60 rpm, bt: 135 °C, m: 35 L/h		0.087 ± 0.005	
		Single; ss: 60 rpm, bt: 142 °C, m: 25 L/h		0.089 ± 0.004	
Niacin	Oats	Single; ss: 0.012 kWhkg^−1^, bt: 152 °C, m: 7%	1.35	100 *	[115]
	Maize	Single; ss: 0.095 kWhkg^−1^, bt: 130 °C, m: 7%	0.63	83 *	
		Single; ss: 0.095 kWhkg^−1^, bt: 152 °C, m: 7%		75 *	
		Single; ss: 0.095 kWhkg^−1^, bt: 160 °C, m: 7%		73 *	
	Maize + dried green peas	Single; ss: 0.095 kWhkg^−1^, bt: 152 °C, m: 7%	1.85	60 *	
Pyridoxine	Oats	Single; ss: 0.012 kWhkg^−1^, bt: 152 °C, m: 7%	0.10	35 *	[115]
	Maize	Single; ss: 0.095 kWhkg^−1^, bt: 130 °C, m: 7%	0.04	86 *	
		Single; ss: 0.095 kWhkg^−1^, bt: 152 °C, m: 7%		100 *	
		Single; ss: 0.095 kWhkg^−1^, bt: 160 °C, m: 7%		100 *	
	Maize + dried green peas (*Pisum sativum* L.)	Single; ss: 0.095 kWhkg^−1^, bt: 152 °C, m: 7%	0.06	18 *	

Twin or single: type of screw; ss: screw speed; bt: barrel temperature; m: moisture; n.d.: not detected; * values expressed in percentage of retention.

**Table 6:** Effect of extrusion process on some antinutrients (enzyme inhibitor and hemagglutinins) of different pulses.

Food Matrix	Extrusion Conditions	Effect of the Extrusion Process								References
		TIA		CIA		α-AIA		HA		
		NE	E	NE	E	NE	E	NE	E	
*Vicia faba* L.	Twin; ss: 100 rpm; m: 25%; bt: 152 °C	4.47 ± 0.21	0.05 ± 0.01	3.56 ± 0.16	1.68 ± 0.19	18.9 ± 1.80	0.00 ± 0.00	49.3 ± 0.00	0.2 ± 0.00	[145]
*Phaseolus vulgaris* L.	Twin; ss: 100 rpm; m: 25%; bt: 156 °C	3.10 ± 0.24	0.43 ± 0.11	3.97 ± 0.16	0.00 ± 0.00	248 ± 4.25	0.00 ± 0.00	74.5 ± 0.00	0.2 ± 0.00	
*Phaseolus vulgaris* L. Pontal	Single; cr: 3:1; ss: 150 rpm; bt: 150 °C m: 20%	4.64 ± 0.03	1.36 ± 0.12	-	-	18.16 ± 2.98	ND	Presence	Absence	[63]
*Phaseolus vulgaris* L. Grafite	Single; cr: 3:1; ss: 150 rpm; BT: 150 °C m: 20%	4.61 ± 0.21	1.41 ± 0.06	-	-	16.08 ± 1.06	ND	Presence	Absence	
Lentil (*Lens culinaris* L. Medik)	Twin; ss: 500 rpm; bt: 160 °C; m: 17%	11.43 ± 0.52	0.36 ± 0.02	-	-	-	-	1.36 ± 0.14	0.0 ± 0.00	[12]
Lentil (*Lens culinaris* L.) + apple fibre+ corn fibre	Twin; ss: 500 rpm; bt: 160 °C; m: 17%	3.51 ± 0.07	0.08 ± 0.00	-	-	-	-	0.67 ± 0.13	0.0 ± 0.00	
Rice (80%) + pea (*Pisum sativum* L.) (20%)	Twin; ss: 900 rpm; bt: 125 °C; m: 2.50 kg/h	1.33	n.d.	1.32	n.d.	-	-	20.41	0.63	[82]
Rice (60%) + pea (*Pisum sativum* L.) (40%)	Twin; ss: 950 rpm; bt: 125 °C; m: 2.50 kg/h	3.11	n.d.	3.04	n.d.	-	-	20.41	5.13	
Rice (80%) + bean (*Pisum vulgaris* L.) (20%)	Twin; ss: 900 rpm; bt: 125°C; m: 2.50 kg/h	4.10 ± 0.09	n.d.	1.97 ± 0.09	n.d.	-	-	-	-	[43]
Rice (60%) + bean (*Pisum vulgaris* L.) (40%)	Twin; ss: 950 rpm; bt: 125 °C; m: 2.50 kg/h	7.83 ± 0.11	n.d.	5.65 ± 0.17	n.d.	-	-	-	-	

TIA: trypsin inhibitor activity (TIU/mg dw); CIA: chymotrypsin inhibitor activity (CIU/mg dw); α-AIA: α-amylase inhibitor activity (IU/100 g; IU/g dw); HA: haemagglutinating activity; NE: non extruded; E: extruded; twin or single: type of screw; ss: screw speed; bt: barrel temperature; m: moisture; cr: compression ratio; n.d.: not detected.
